# Anaesthetic implications of paediatric thoracoscopy

**DOI:** 10.4103/0972-9941.15240

**Published:** 2005-03

**Authors:** Nandini Dave, Sarita Fernandes

**Affiliations:** Department of Anaesthesiology, BYL Nair Hospital & TN Medical College, Mumbai - 400008, India

**Keywords:** Thoracoscopy, anaesthesia, paeditaric

## Abstract

Anaesthetic care during thoracic surgical procedures in children combines components of the knowledge bases of paediatric anaesthesia with those of thoracic anaesthesia. This article highlights the principles of anaesthesia during thoracoscopic surgery in children including preoperative evaluation, anaesthetic induction techniques, maintenance anaesthesia and options for postoperative analgesia. In addition, given the need to provide optimal surgical visualization during the procedure, one lung ventilation may be required. Techniques to provide one lung ventilation in the paediatric patient and the principles of anaesthesia care during one lung ventilation are discussed.

## INTRODUCTION

Thoracoscopy in children initially was proposed as a method of obtaining pulmonary biopsy specimens in immunocompromised patients. With further refinements in the technique and development of better instrumentation, the scope has widened tremendously with more complicated procedures like PDA ligation, thymectomy, Heller’s myotomy, congenital diaphragmatic hernia repair etc. being performed with the help of thoracoscopy.

Anaesthesia for paediatric thoracoscopy[1,2] is very challenging as the paediatric anaesthesiologist has to be well versed in the various techniques of providing one lung anaesthesia and manage the intra and postoperative complications. Utmost vigilance is needed as one encounters arrhythmias (such as ventricular tachycardia, atrial fibrillation, supraventricular extrasystoles etc.), mediastinal shift, hypertension or hypotension and hypercapnia. Pulmonary complications include hypoxemia, hypercarbia, impaired hypoxic pulmonary vasoconstriction, re-expansion pulmonary oedema, atelectasis and pneumonia. There is always the possibility of some major vessel injury and torrential bleed. It is difficult to assess the blood loss during thoracoscopy.

## PRE-OPERATIVE EVALUATION

Patients presenting for thoracoscopic surgery should undergo a similar preoperative evaluation to those presenting for open thoracotomy with special emphasis on the degree of pulmonary and cardiac dysfunction. It is customary to obtain a complete history, physical examination and the following laboratory tests: haemoglobin, haematocrit, liver function tests, serum electrolytes and an X-ray Chest. Additional preoperative evaluations such as pulmonary function test (PFT) and ECG are not routinely indicated but rather obtained based on the patients medical history and associated underlying illness. Preoperative CT scan of the chest is useful in children with an anterior mediastinal mass. Compression of greater than 50% of the cross sectional area of the trachea on CT imaging can be used to identify the high risk population in whom general anaesthesia with loss of spontaneous ventilation can lead to total airway obstruction. Options include preoperative radiation or chemotherapy to shrink the mass or induction of general anaesthesia while maintaining spontaneous ventilation with cardiopulmonary bypass as a backup measure. Simple bedside spirometry—FVC, FEV1 and the ratio FVC/FEV1 may be performed in older children to assess the degree of obstructive lung disease and ensure that the minimum criteria for wedge or lung resection are satisfied.

## PRE-OPERATIVE PREPARATION

Chest physiotherapy, good nutrition, bronchodilator/antibiotic therapy, steroid supplementation etc. helps in optimizing the patients condition prior to surgery.

As there is always a possibility of conversion to open thoracotomy, blood should be kept in reserve.

Standard perioperative monitoring includes

1. ECG
2. Pulse oximetry
3. End tidal CO_2_ measurement
4. Noninvasive BP monitoring
5. Continuous temperature monitoring.

The bladder is catheterised and urine output monitored when surgery is prolonged or significant blood loss expected.

### Premedication and anaesthesia management

In otherwise healthy patients without airway compromise intranasal Midazolam 0.3 mg/kg in children without intravenous access or rectal or oral Midazolam 0.5-0.75 mg/kg administered 15 to 20 minutes prior to anaesthesia induction provides anxiolysis, easy separation from parents and acceptance of face mask.

Blood loss during a diagnostic thoracoscopy is usually minimal. It is however advisable to have preferably two venous accesses prior to the start of the procedure as the surgery is performed in the lateral decubitus position.

If central venous pressure monitoring is necessary, internal or external jugular monitoring on the side of thoracoscopy is recommended. In patients with severe cardiac instability and where major haemodynamic fluctuations are expected, invasive arterial blood pressure monitoring is used. Atropine is administered as a vagolytic and antisialogogue. Antiemetics and H2 antagonists are administered in patients at risk for aspiration.

Inhalational Sevoflurane or Halothane or intravenous Thiopentone or Propofol induction is followed by a neuromuscular blocking drug to facilitate endotracheal intubation.

Intraoperative analgesic used is generally Fentanyl 1-2 microgram/kg or Pentazocine 0.6 mg/kg,

Anaesthesia is maintained either by using inhalational agents or infusions of Propofol. Patient is maintained on controlled ventilation using short acting muscle relaxants.

The goals of anaesthesia include: minimizing airway reactivity, optimizing gas exchange, maintaining stable cardiovascular function, preventing ventilatory depression and providing adequate pain relief in the postoperative period.

### Anaesthesia technique for thoracoscopy

A variety of anaesthesia techniques can be used for thoracoscopy. Older children (> 8 years of age or weight > 30-35 kg) can be managed using most of the techniques used in adults. Special techniques for isolation of the operative lung are suitable for smaller children.

1. Local anaesthesia- may be possible in older adolescents. This technique is usually reserved for brief procedures without involved intrathoracic surgical manipulation for ill patients with unacceptable risk of perioperative morbidity following general anaesthesia. Following IV sedation, the lateral chest wall and parietal pleura are infiltrated with local anaesthetic to provide anaesthesia for trocar placement.
2. Regional techniques include thoracic epidural anaesthesia, thoracic paravertebral blocade, multiple intercostal blocks or intrapleural analgesia. The stellate ganglion block also temporarily eliminates the cough reflex which can be elicited during manipulation of the pulmonary hilum.
  Regional anaesthesia techniques and local anaesthesia with sedation offers the advantage of maintaining spontaneous ventilation and interferes less with surgical exposure. However patients with significant pulmonary disease are sometimes unable to compensate for the temporary loss of pulmonary surface area due to partial collapse of the lung on the side of the thoracoscopy.
  Local and regional techniques are possible only in the older age group. In majority of cases thoracoscopy is always performed under general anaesthesia with lung isolation techniques whenever feasible.
3. General anaesthesia and one lung ventilation: With general anaesthesia and positive pressure ventilation, intrathoracic visualization and surgical access can be impaired by lung movement. To overcome this problem, thoracoscopy is performed using techniques to isolate the lung and provide one lung ventilation. This allows the lung on the operative side to be collapsed and motionless, facilitating exposure and surgical instrumentation, while gas exchange (oxygenation and CO_2_ elimination) is maintained by ventilating the non-operative dependant lung.

Techniques for one lung ventilation in children.[3](Table 1)

**Table 1:** Tube selection for single lung ventilation in children[3]

Age	ETT (ID)	BB (Fr)	Univent	DLT (Fr)
0.5-1		3.5-4	2	
1-2	4-4.5	3		
2-4	4.5-5	5		
4-6	5-5.5	5		
6-8	5.5-6	5	3.5	
8-10	6 cuffed	5-7	3.5	26
10-12	6.5 cuffed	7	4.5	26-28
12-14	6.5-7 cuffed	7	4.5	32
14-16	7 cuffed	7-9	6	35
16-18	7-8 cuffed	9	7	35

1. Selective mainstem intubation
2. Double lumen endotracheal tube (DLT)
3. Bronchial blockers
4. Univent endotracheal tube

**Figure 1:**
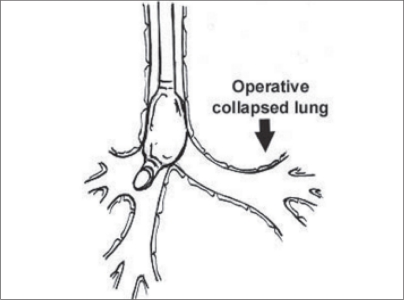
Right mainstem endobronchial intubation

**Figure 2:**
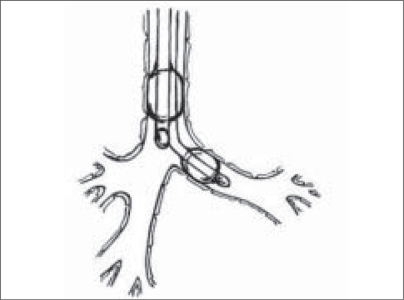
Left sided DLT

**Figure 3:**
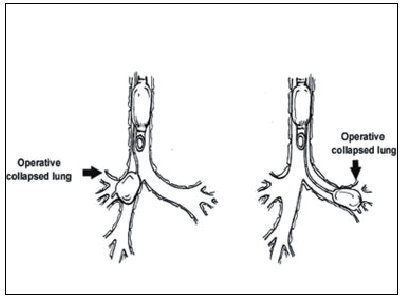
Bronchial blockers

1. **Selective mainstem intubation** is a simple and quickly achieved means of one lung ventilation in patients whose small size precludes placement of a DLT or Univent tube. The tracheal tube should be one half smaller than usual, based on patient’s age as the diameter of the mainstem bronchus is smaller than that of the trachea. Bronchoscopic guidance or fluoroscopy can aid correct placement. As an uncuffed tracheal tube might not be totally occlusive to avoid soilage and inadvertent ventilation of the operative side, a cuffed tracheal tube is recommended in patients > 2 years of age (Figure 1).
2. **Double lumen tube**
  When patient size permits, a DLT is preferable as it has advantages over other techniques.
  1. Rapidly and easily separating the lungs
  2. Allowing for suctioning of both lungs
  3. Providing rapid switch to two lung ventilation as necessary based on patients status.
  4. Improving oxygenation by applying CPAP to the operative lung and PEEP to the non-operative lung.
  In children left sided DLTs are used almost exclusively because they are easier to place and eliminate concern of obstruction of right upper lobe bronchus.
  However specialized paediatric bronchoscopes are needed to confirm correct placement. As the smallest commercially available DLT is a 26 Fr tube, placement in patients weighing less than 30-35kg or younger than 8 -10 years of age is not feasible (Figure 2).
3. **Bronchial Blockers**
  The bronchial blocker eg. Fogarty embolectomy catheter[4] (Table 2), Swan-Ganz catheter or Arndt bronchial blocker can be placed in the mainstem bronchus of the operative side blindly, using X-ray guidance or under direct vision with a fibreoptic bronchoscope. All the various devices have a balloon at the end that is inflated to occlude the bronchus of the operative lung.
  With an inflated blocker balloon, the airway is completely sealed, providing more predictable lung collapse and better operating conditions than with an endotracheal tube in the bronchus. Those devices with a central channel provide the advantage of allowing some degree of suctioning through the channel, not to clear the lung of secretions (the channel is too small for that purpose) but rather to deflate the operative lung or for application of continuous positive airway pressure.
  A potential problem is dislodgement of the blocker balloon into the trachea. The inflated balloon will then block ventilation to both lungs. When closed tip blockers are used, the operative lung cannot be suctioned, the lung may not deflate completely and continuous positive airway pressure cannot be provided to the operated lung if needed.(Figure 3)
4. **Univent tube**
  Univent tube is a single lumen tracheal tube with a movable bronchial blocker that is incorporated into a channel placed alongside the tube.
  Advantages of the Univent tube include easy placement, the ability to change intermittently from one to two lung ventilation and a channel through the bronchial blocker that permits oxygen insufflation into the operative lung during one lung ventilation. DLTs merely separate right from the left whereas with the Univent tube, there is a facility to block selectively a lobar or even a segmental bronchus. Since the bronchial blocker is incorporated into the tube, displacement is less likely.
  Disadvantages: The large amount of cross sectional area occupied by the blocker channel causes high resistance to ventilation. The low volume, high pressure characteristics of the blocker balloon can result in mucosal injury.
  The Univent tube is marketed in 0.5 mm increments from ID 6 mm to 9 mm. Now paediatric sizes 3.5 and 4.5 mm ID are also available. Univent tubes require fibreoptic bronchoscope for successful placement.
  Paediatric bronchoscopes are 3.5 -4 mm in diameter and slighter shorter than adult bronchoscopes. An ultrathin bronchoscope 2.2 mm in diameter is also available and can be used with the smallest of endotracheal tubes. In units where paediatric fibrescopes are not available, rigid bronchoscopy may be required to accurately position bronchial blockers.(Figure 3)

**Table 2:** Fogarty catheter size for lung isolation in children by age[4]

Age (y)	Size of Fogarty Catheter (F)	
	For boys	For girls
0-4	3	3
4-10	4 or 5	4 or 5
10-12	4 or 5	5
>12	5 or 6	6

### Perioperative management

Physiology of One Lung Ventilation (OLV)[5]

OLV provides excellent surgical conditions and is associated with a low incidence of accidental lung injury. After the endobronchial tube is placed CO2 insufflation augments lung collapse and provides additional lung protection during the insertion of endoscopic instruments.

Following partial lung collapse, hypoxic pulmonary vasoconstriction (HPV) increases pulmonary vascular resistance with consequent re-routing of blood to the well ventilated lung zones. This normal physiologic response to atelectasis decreases ventilation perfusion mismatching and improves arterial oxygenation. However, when more than 70% of the lung is atelectatic (as in OLV), HPV is obliterated.

After collapse of the operative upper lung, all the ventilation passes to the lower lung, but blood flow though reduced persists in the nonventilated lung. This blood flow does not participate in oxygenation and represents a right to left transpulmonary shunt which accounts for the decrease in arterial PO2 seen during OLV. Preexisting pulmonary disease, elevation of the diaphragm, compression of the thoracic cavity from the mediastinum, abdominal contents, rolls and packs used to facilitate positioning of the patient, all contribute to a disparity between ventilation and perfusion in the ventilated lung thus increasing the shunt fraction still further.

The physiologic basis for haemodynamic instability during OLV is multi-factorial. As the chest cavity is closed, rapid and excessive CO2 insufflation can create a tension pneumothorax. The resultant compression of the lungs and great vessels could decrease venous return and stroke volume with resultant hypotension. Gas insufflation can activate pulmonary stretch receptors and increase vagal tone with consequent bradycardia and also can cause mediastinal shifting and cardiac tamponade.

### Methods to improve oxygenation

1. High FiO_2_—first line of therapy in c/o hypoxemia
2. Tidal volume—8-12 ml/kg to the ventilated lung prevents atelectasis
3. Airway pressure if too high—Tidal volume may be decreased and respiratory rate increased.
4. CPAP to the operative lung
5. PEEP to the ventilated lung[6]
6. High frequency jet ventilation at low driving pressures 10-12psi to the operative lung
7. Ipsilateral pulmonary artery can be clamped
8. Re-inflation of non-ventilated lung (prophylactically every 5 mins)
9. Maintain cardiac output

CO_2_ insufflation[7] into the operative hemithorax is used as a technique to facilitate collapse of the lung on the operative side. This is particularly useful in smaller patients where lung isolation is not possible and there is inadequate separation of the two lungs with overflow ventilation into the operative side. Meticulous cardiopulmonary monitoring is mandatory as displacement of intrathoracic contents and creation of an excessive pneumothorax can lead to significant cardiovascular compromise from decreased venous return or high left ventricular afterload. The effects of the artificial pneumothorax can be minimized by slowly adding the CO_2_ (flow rate 1 L/min) and limiting the inflating pressure to 4 to 6 mmHg. Direct insufflation of CO_2_ into the lung parenchyma can cause sudden rise in End Tidal CO_2_. Subcutaneous emphysema and CO_2_ embolism can occur. Detection techniques for gas embolism that can identify the problem prior to the onset of cardiovascular changes include transoesophageal Echo (0.1 ml of gas), precordial doppler (0.5 ml) and capnometric end tidal Nitrogen monitoring. The combination of standard tracheal intubation with prone position and CO_2_ insufflation may provide good exposure in some cases.[8]

Once successful separation of the non-operative and operative lung has been accomplished, anaesthesia is maintained with a combination of intravenous and inhalational anaesthetics. Isoflurane (MAC limited to 0.5-1.0 MAC) preserves HPV. Fentanyl, Ketamine, Benzodiazepines and Barbiturates have little or no effect on HPV. Any non-specific vasodilation (eg. Terbutaline, Albuterol, Isoproterenol, Dobutamine, Nitroglycerine and Sodium nitroprusside) can impair HPV and affect oxygenation during one lung ventilation.

### Post-operative complications[9]

1. Persistent air leak is the most common complication following VATS which can lead to subcutaneous emphysema, residual pnemothorax or recurrent pneumothorax.
2. Down Lung syndrome is the term for increased secretions and pneumonia that can develop postoperatively in either lung following OLV.
3. Infection ranges from a local wound infection to a pulmonary abcess or empyema.
4. Horner syndrome
5. Dissemination of malignant disease
6. Lung herniation through the chest wall.
7. Recurrent laryngeal nerve injury is seen more with mediastinoscopy. If suspected, possibility of airway obstruction should be kept in mind.

### Post-operative pain control[10]

Thoracoscopic procedures offer the advantage of small incisions without either splitting of the serratus anterior or latissimus dorsi muscles or spreading of the ribs, two techniques which markedly contribute to postoperative pain. In order to minimize pain, patients breathe rapidly with small tidal volumes. This type of breathing promotes atelectasis, retention of secretions, decrease in functional residual capacity and increase in V/Q mismatching all of which contribute to hypoxemia. Good pain relief by any method is mandatory.

1. Oral: Nonsteroidal anti-inflammatory drugs, Paracetamol etc
2. Rectal suppositories: Paracetamol and Diclofenac provide long lasting analgesia and reduce opioid requirements.
3. Intravenous route: Most patients especially those who have undergone pleural procedures such as decortication, pleurectomy or pleurodesis require potent parenteral opiods for the first 24 hours. IV opiods via PCA pump using Morphine or Fentanyl provide satisfactory analgesia especially in older children who are able to comprehend and express pain.
4. Intercostal nerve blocks or intrapleural installation of Bupivacaine relieves pain from chest tubes or instrument insertion points.
5. Epidural analgesia:The administration of neuraxial opiods or local anesthetics through an epidural catheter is usually unnecessary after a straightforward thoracoscopy but is almost always inserted if the thoracoscopic procedure is converted to an open thoracotomy.

In our setup we have found that rectal suppositories, IV Tramadol (1-2 mg/kg) 6-8hrly along with local infiltration of 0.25% Bupivacaine at the port sites provides satisfactory analgesia following thoracoscopy.

## CONCLUSION

In conclusion, minimal access surgery does not mean minimally invasive anaesthesia. A thorough knowledge of the physiology of one lung ventilation, meticulous planning, continuous vigilance to detect any untoward event at the earliest and good communication between the anaesthesiology and surgical teams all contribute to a safe and successful surgery.
